# Identification of metabolomic changes and potential therapeutic targets during ovarian aging

**DOI:** 10.18632/aging.206119

**Published:** 2024-10-09

**Authors:** Bo Sun, Lu Li, Xiaoli Chen, Yingpu Sun

**Affiliations:** 1 Center for Reproductive Medicine, The First Affiliated Hospital of Zhengzhou University, Zhengzhou 450052, Henan Province, China; 2 Henan Key Laboratory of Reproduction and Genetics, The First Affiliated Hospital of Zhengzhou University, Zhengzhou 450052, Henan Province, China

**Keywords:** metabolomics, ovarian aging, GABA, succinic acid, ART

## Abstract

Purpose: To reveal the metabolic differences of follicle fluid (FF) and granulosa cell (GC) between younger women and advanced age women in ART cycles, and then find potential therapeutic targets of ovarian aging.

Methods: Forty-five patients were included in the study and they were divided into three groups according to their age (Group A: 20–30 years old; Group B: 30–35 years old; Group C: 35–45 years old). All patients underwent controlled ovarian stimulation using the follicular phase long-acting protocol, FF and GC were obtained 36–38 hours after HCG administration. Liquid chromatography-tandem mass spectrometry (LC-MS/MS) was used for metabolomics analysis and metabolic pathway analysis (MetPA) was utilized to find related pathways.

Results: Between group A and group C, there were 72 and 21 differential metabolites in FF and GC, respectively. KEGG enrichment analysis showed six pathways were co-enriched by the differential metabolites of FF and GC. Among them, we noticed that in the pathway GABAergic synapse, GABA (gamma-aminobutyric acid) was down-regulated in GC, while its downstream metabolite succinic acid was down-regulated in FF. Further ROC curve analysis was performed on these two metabolites, and the results showed that they all had a favorable predictive value.

Conclusion: This study indicated that GABA and succinic acid could be potential therapeutic targets for ovarian aging, GABA may delay ovarian aging and improve ovarian function through its antioxidant properties, which may be a future direction of clinical treatment.

## INTRODUCTION

As an independent risk factor of female fertility, age has always been a top issue for discussion. The fecundability of women declines significantly after their mid −30 s [1], many scholars consider 35 years as the cut-off for reproductive age, and childbearing women over 35 years were defined as advanced maternal age (AMA) [1, 2]. Currently, there are no remedies to counteract the fertility decline associated with aging, even using assisted reproductive technology (ART), AMA also seriously impairs its success rate [1]. As patients get older, the ovarian reserve diminishes and their response to exogenous gonadotropins decreases [3], while egg quality degrades and the aneuploidy rate increases notably [4], all of which lead to a reduction in the live birth rate and an increase in the miscarriage rate in AMA [5]. How to improve ART outcomes and reproductive health in elderly patients is a practical problem that urgently needs to be solved.

Oocyte quality and quantity are the major limiting factors for female success in ART, GCs could provide nutrients to oocytes and control the transcriptional activity of development-related genes, thereby regulating oocyte development [6, 7]. As GC decreases with age, the proteins, metabolic secretions, hormones, and ions they secrete may be altered, directly affecting the oocyte or causing changes in the microenvironment that are essential for follicle growth and development and oocyte maturation. Moreover, GCs and oocytes are both exposed in FF, while the latter provides a specific microenvironment for oocyte development and maturation [8]. FF contains a variety of components; these molecules have a role in regulating oocyte and follicle maturation and protecting GCs from physical or oxidative damage [9]. In-depth study of the metabolic changes of GC and FF during aging can provide a better understanding of the effects of advanced age on female reproduction and provide directions for the development of new therapeutic methods and ideas in clinical practice.

Metabolomics is an emerging technology in recent years, that can detect metabolic alterations in the body to identify disease-related metabolic disorders, and is widely used in many disciplines, including reproductive medicine, because of its high resolution, sensitivity, and throughput [10]. Song et al. assayed serum metabolites in females with normal ovarian reserve and poor ovarian response, and revealed that the nicotinate and nicotinamide metabolic pathways are vitally important [11]. Similarly, Yu et al. investigated the differences in serum metabolomics of patients with polycystic ovary syndrome (PCOS) [12]. Applying liquid chromatography-tandem mass spectrometry (LC-MS/MS) in this study, we analyzed the changes of aging on metabolites of GCs and FF in detail to elucidate and resolve the changes in cellular signal and metabolic network caused by aging. The refined analysis of metabolite changes will help to explore female reproductive aging biomarkers as well as provide promising insights for improving the effects of aging on granulosa cells.

## MATERIALS AND METHODS

### Study population and sample collection

A total of 45 women were included in this study. The participants had undergone the first ART cycles. All the participants included in the study have provided written informed consent, and their cause of infertility was all tubal obstruction, patients with PCOS, endometriosis, premature ovarian insufficiency, and chromosome abnormality were excluded.

Follicular phase long-acting protocol: patients were injected 3.75 mg GnRH antagonist (GnRH-a Tryptorelin, Ferring, Germany) on the 2nd day of menstruation if the ultrasound did not find cysts and follicles >10 mm. The patients will visit the hospital 28 days after the injection to be examined on ultrasound+serum FSH, LH, E2, and progesterone (P). Administration of a starting dose of 150–300 IU of human recombinant FSH (rFSH, Gonal F, Serono Ltd., Switzerland), with subsequent adjustment of gonadotropin (Gn) use according to follicular growth. The starting dose is according to age, AFC, and basal hormone levels. Follicular development was detected by using vaginal ultrasound. Ovulation is performed using human chorionic gonadotropin (HCG) (2,000 IU) (Lizhu Ltd., China) and recombinant HCG (250 µg) (Serono Ltd., Switzerland) when at least one follicle was 18 mm in size, and FF and GCs were obtained 36–38 hours after HCG administration.

Depending on their age, patients were divided into three groups (Group A: 20–30 years old; Group B: 30–35 years old; Group C: 35–45 years old). Briefly, follicular aspirates were centrifuged at 1600 RMP for 15 minutes, the supernatant was FF and the sediment was the cell mixture. The purified GCs were obtained by density gradient centrifugation of the sediment as previously described [13]. Both FF and GCs were stored at −80°C until assayed.

### Metabolite extraction method

GCs: add 100 µl of the whole target isotope internal standard to a 2 ml EP tube containing 1 × 10^7^ cells and vortex for 30 s to mix the cells. Then add 400 µl of acetonitrile: (methanol: ddH_2_O mixed solution (2:2:1, V:v:v)) and vortex oscillation for 30 s. Place the centrifuge tube containing the sample, invade liquid nitrogen for rapid freezing for 5 minutes, take out the centrifuge tube, and freeze-thaw at room temperature on the double panel. Place the centrifuge tube in the 2 ml adapter again, and install it until grinding in the instrument. Oscillate at 60 Hz for 2 min. Repeat the last step twice, take out the centrifuge tube, centrifuge at 12000 rpm 4°C for 10 min, take 450 µl of the supernatant into a 2 ml centrifuge tube, and concentrate in vacuo to dryness. Accurately add 100 µl of acetonitrile (0.1% FA (1:9, v:v) solution (without 2-chlorophenylalanine)) (stored at −20°C) to reconstitute the sample, filter through a 0.22 µm membrane, and add the filtrate to detection bottle.

FF: thaw all samples at 4°C. Transfer 100 µL of each sample into 2 mL centrifuge tubes. Add 100 µL of mixed internal standard solution and 400 µL of methanol (−20°C), then vortex for 60 s. Centrifuge at 4°C for 10 min at 12000 rpm, and then transfer 500 µL of the supernatant from each sample into another 2 mL centrifuge tube. Samples were concentrated to dry in vacuum. Dissolve samples with 150 µL of 80% methanol solution, and centrifuge at 4°C for 10 min at 12000 rpm again to obtain the supernatant for LC-MS.

After the above procedure, 20 µL of each sample was used for quality control (QC), while the rest of the samples were then used for LC-MS analysis.

### LC-MS/MS analysis

Chromatographic separations were performed on a Thermo Ultimate 3000 system equipped with an ACQUITY UPLC^®^HSS T3 (150 × 2.1 mm, 1.8 µm, Waters, Milford, MA, USA) column maintained at 40°C. The autosampler temperature was 8°C. The analytes were eluted at 0.25 ml/min with a gradient of 0.1% formic acid solution (C) and 0.1% acetonitrile formatted solution (D) or 5 mm ammonium formatted solution (A) and acetonitrile solution (B). After equilibration, 2 µl was injected into each sample. The linear-gradient (v/v) of solvent B increased: 0–1 min, 2% B/D; 1–9 min, 2–50% B/D; 9–12 min, 50–98% B/D; 12–13.5 min, 98% B/D; 13.5–14 min, 98–2% B/D; 14–20 min, 2% d positive model (14–17 min, 2% b negative model).

ESI-MSn experiments were performed on a Thermo Q Exactive HF-X mass spectrometer using spray voltages of 3.5 kV and −2.5 kV in positive and negative modes, respectively. The casing and auxiliary gases were arbitrary units of 30 and 10, respectively. The capillary temperature was 325°C. The analyzer was fully scanned in the mass range m/z 81–1,000 with a mass resolution of 60,000. Data dependent acquisition (DDA) MS/MS experiments were performed using HCD scans. The normalized collision energy was 30 eV. A dynamic exclusion method was used to remove unwanted information from the MS/MS spectrum.

### Statistical methods and bioinformatic analysis

The baseline and ovarian stimulation characteristics of patients were analyzed. Mann–Whitney tests were used for continuous variables and categorical variables were examined by Chi-squared tests. The results are shown as the mean ± standard deviation (SD) or % (n/N). All data were analyzed by SPSS 23.0 (IBM Corp., NY, USA), and the threshold was set as a two-tailed *P* < 0.05.

Comprehensive metabolomic data analysis was performed by R-software (Metabo Analyst 3.0). Firstly, sample normalization of the metabolomics data was performed. Principal component analysis (PCA) of metabolomics data was performed on a normalized dataset. Analysis of variance (ANOVA) was used to analyze the metabolites among the three groups, a *P*-value < 0.05 was considered as the differential metabolites. Next, Partial Least Squares Discriminant Analysis (PLSDA) was performed in Group A and Group C to decipher differences between them, and Variable Importance Projection (VIP) scores were generated in Metabo Analyst. To identify different metabolites between groups, the threshold was set as VIP ≥ 1, |Log2FC (fold change) |> 1 or <−1 and *P*-value < 0.05. Metabolic pathway enrichment analysis of the identified metabolites was carried out in the Kyoto Encyclopedia of Genes and Genomes (KEGG) database.

### Availability of data and materials

All data generated or analyzed during this study are included in this published article (and its Supplementary Tables).

## RESULTS

### Baseline characteristics and ovarian induction characteristics of patients

As shown in Table 1, no significant differences were found among the three groups in body mass index (BMI) (22.41 ± 0.48 vs. 21.51 ± 0.45 vs. 23.87 ± 0.55), basal serum FSH (5.87 ± 0.54 vs. 6.66 ± 0.42 vs. 6.40 ± 0.34), basal serum LH (5.21 ± 0.70 vs. 3.51 ± 0.76 vs. 4.38 ± 0.50), AMH (4.35 ± 0.98 vs. 3.16 ± 0.60 vs. 2.75 ± 0.48), AFC (16.53 ± 0.68 vs. 12.93 ± 1.37 vs. 11.80 ± 1.54) and basal endometrial thickness (5.05 ± 1.03 vs. 5.71 ± 1.07 vs. 5.94 ± 1.25).

**Table 1:** Baseline characteristics and ovarian induction characteristics of patients.

	**Group A (20–30y)**	**Group B (30–35y)**	**Group C (35–45y)**	***P*-value**
Female age	26.80 ± 2.08	32.27 ± 1.16	37.27 ± 1.83	^#^0.002^※^0.005*0.000
BMI	22.41 ± 1.87	21.51 ± 1.75	23.87 ± 2.12	0.136
Type of infertility				0.001
Primary infertility	12	11	3	
Secondary infertility	3	4	12	
Basal FSH (mIU/mL)	5.87 ± 2.09	6.66 ± 1.64	6.40 ± 1.32	0.698
Basal LH (mIU/mL)	5.21 ± 2.73	3.51 ± 2.96	4.38 ± 1.96	0.294
AMH (ng/mL)	4.35 ± 3.78	3.16 ± 2.34	2.75 ± 1.86	0.672
Basal endometrial thickness (mm)	5.05 ± 1.03	5.71 ± 1.07	5.94 ± 1.25	0.833
Type of ART				1.000
IVF	14	14	14	
ICSI	1	1	1	
Starting dosage of Gn (IU)	151.67 ± 63.01	194.17 ± 66.46	212.50 ± 51.97	*0.006
Duration of stimulation (d)	12.20 ± 1.37	13.73 ± 1.79	13.80 ± 2.48	0.059
Total amount of Gn (IU)	2116.67 ± 908.60	3058.33 ± 1102.01	3525.00 ± 1110.12	^※^0.030*0.002
Endometrial thickness on HCG (mm)	12.67 ± 2.09	12.33 ± 1.94	12.00 ± 2.90	0.799
Hormone levels on HCG day				
E2 (pg/mL)	3460.40 ± 1776.59	3608.40 ± 1361.00	2601.79 ± 1467.61	0.082
LH (mIU/mL)	1.05 ± 0.77	0.98 ± 0.71	0.87 ± 0.40	0.929
P4 (ng/mL)	1.09 ± 0.52	1.19 ± 0.44	0.99 ± 0.46	0.611
No. of ≥14 mm oocytes	9.00 ± 2.36	7.60 ± 1.55	7.93 ± 2.19	0.254
No. of oocytes retrieved	15.53 ± 6.28	14.60 ± 4.22	10.33 ± 5.01	^※^0.037*0.011
No. of MII oocytes	13.40 ± 4.97	12.87 ± 3.96	8.93 ± 4.57	^※^0.020*0.011
No. of 2PN fertilization	9.80 ± 3.05	9.20 ± 3.99	6.33 ± 3.83	*0.014
No. of transferable embryo	6.20 ± 2.46	5.67 ± 2.50	3.87 ± 2.39	*0.013
No. of good-quality embryo	6.13 ± 2.56	5.33 ± 2.44	3.87 ± 2.39	0.066
ET rate	60% (9/15)	80% (12/15)	87% (13/15)	0.311
Biochemical pregnancy rate	89% (8/9)	75% (9/12)	62% (8/13)	0.438
Clinical pregnancy rate	89% (8/9)	67% (8/12)	62% (8/13)	0.411
Live birth rate	78% (7/9)	50% (6/12)	54% (7/13)	0.457
Abortion rate	12.5% (1/8)	25% (2/8)	12.5% (1/8)	1.000

Numbers are mean ± standard deviation or N (% of response group). Abbreviations: FSH: follicle-stimulating hormone; E2: estradiol; LH: luteinizing hormone; BMI: body mass index; AMH: anti-Mullerian hormone; ART: assistant reproductive technology; Gn: gonadotropin; on HCG day: on the day of human chorionic gonadotropin administration; MII: mature oocytes; 2PN: fertilized oocytes with two pronuclei; ET: embryo transfer. ^#^Group A vs. Group B; ^※^Group B vs. Group C; ^*^Group A vs. Group C.

Compared with group B, group A was associated with a lower total amount of gonadotropin (Gn), more MII oocytes, and more 2PN embryos. Compared with group C, patients in group A used fewer starting dosages of Gn (151.67 ± 16.27 vs. 212.50 ± 13.42), fewer total amount of Gn (2116.67 ± 234.60 vs. 3525.00 ± 286.63), more retrieved oocytes (15.53 ± 1.62 vs. 10.33 ± 1.29), more MII oocytes (13.40 ± 1.28 vs. 8.93 ± 1.18), more 2PN embryos (9.80 ± 0.79 vs. 6.33 ± 0.99) and more transferable embryos (5.78 ± 0.74 vs. 4.31 ± 0.61).

### Profile of GC and FF metabolomics

**Figure 1:**
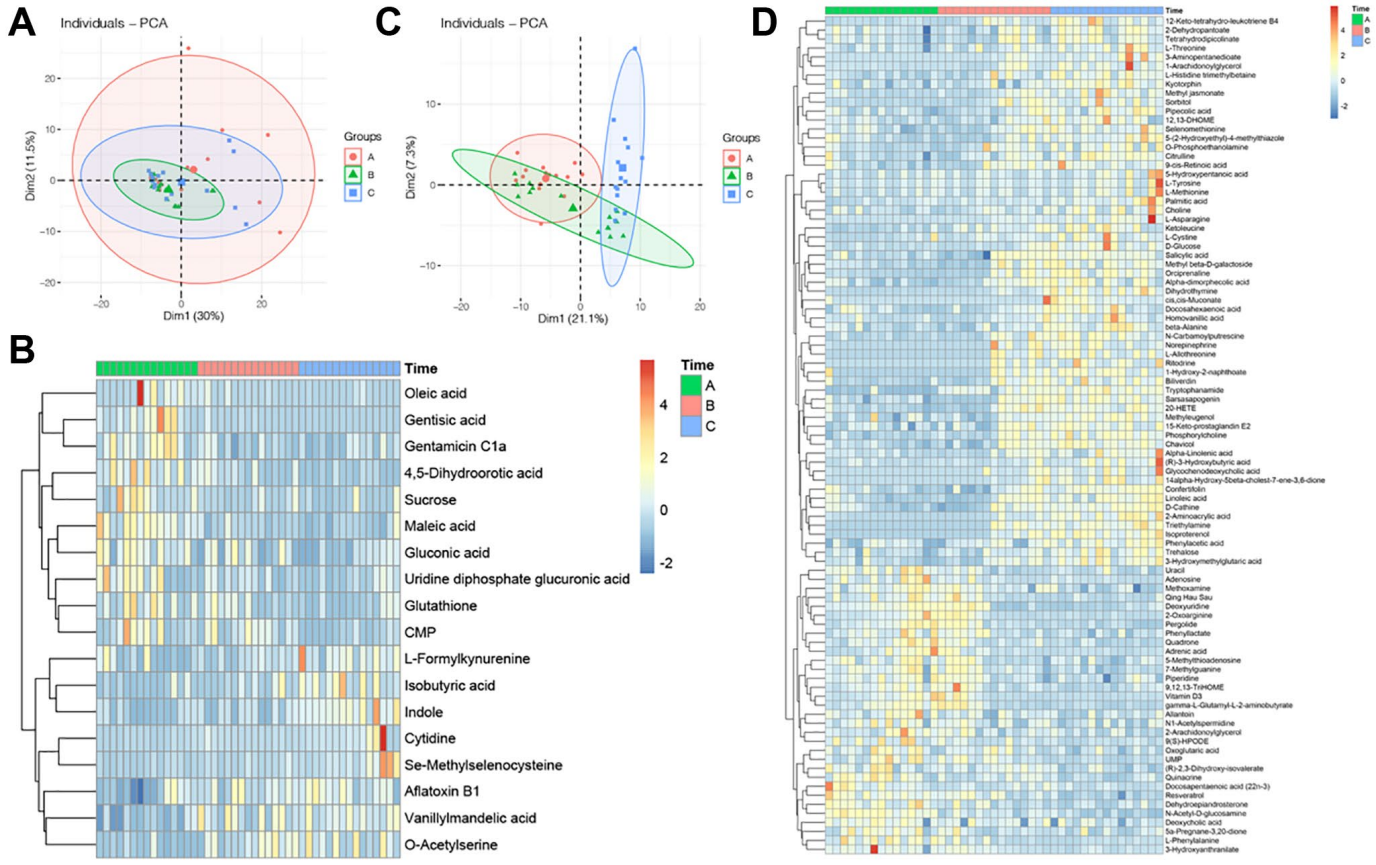
**Profile of GC and FF metabolomics.** (**A**) PCA scatter plot of metabolomics data from FF in three groups. Each point represents one sample, different groups are marked with different colors, and the area marked by the ellipse is the 95% confidence region of the sample points; (**B**) heatmap of hierarchical cluster analysis of FF metabolites, the former ten metabolites decreasing with aging, while the latter eight metabolites increasing with aging. The magnitude of relative content in the plot is shown by the difference in color, where the columns represent samples and the rows represent metabolites; (**C**) PCA scatter plot of metabolomics data from GC in three groups; (**D**) heatmap of hierarchical cluster analysis of FF metabolites, the former 61 metabolites increasing with aging, while the latter 32 metabolites decreasing with aging.

After the normalization of the intensity of metabolites, PCA was performed. The PCA scatter plot of metabolomics data from the FF in the three groups showed that there was a cluster between Group A and Group C, but did not differ significantly between Group A and Group B (Figure 1A). Then, we performed a hierarchical clustering analysis of the screened differential metabolites in all the groups, distinguishing them by different colors. Regions with similar colors represent that metabolites in that region have similar functions or participate in a biological process together, while larger color differences indicate that the functions of metabolites in these regions are far apart. Here, 93 metabolites showed gradual variations in different groups, with 61 gradually elevating and 32 gradually declining (Figure 1B).

The PCA plot reflected the original state of GC metabolites, it showed that the three groups cannot be completely separated (Figure 1C). To investigate the differences in GC metabolites at different ages, ANOVA analysis was performed on 3 sets of metabolite data to screen for metabolites with significant differences, and the clustering of metabolites was visualized by heatmap, results indicated that there were ten metabolites declined with aging, while eight metabolites increased with aging (Figure 1D).

### Metabolic differences in GC between group A and group C

**Figure 2:**
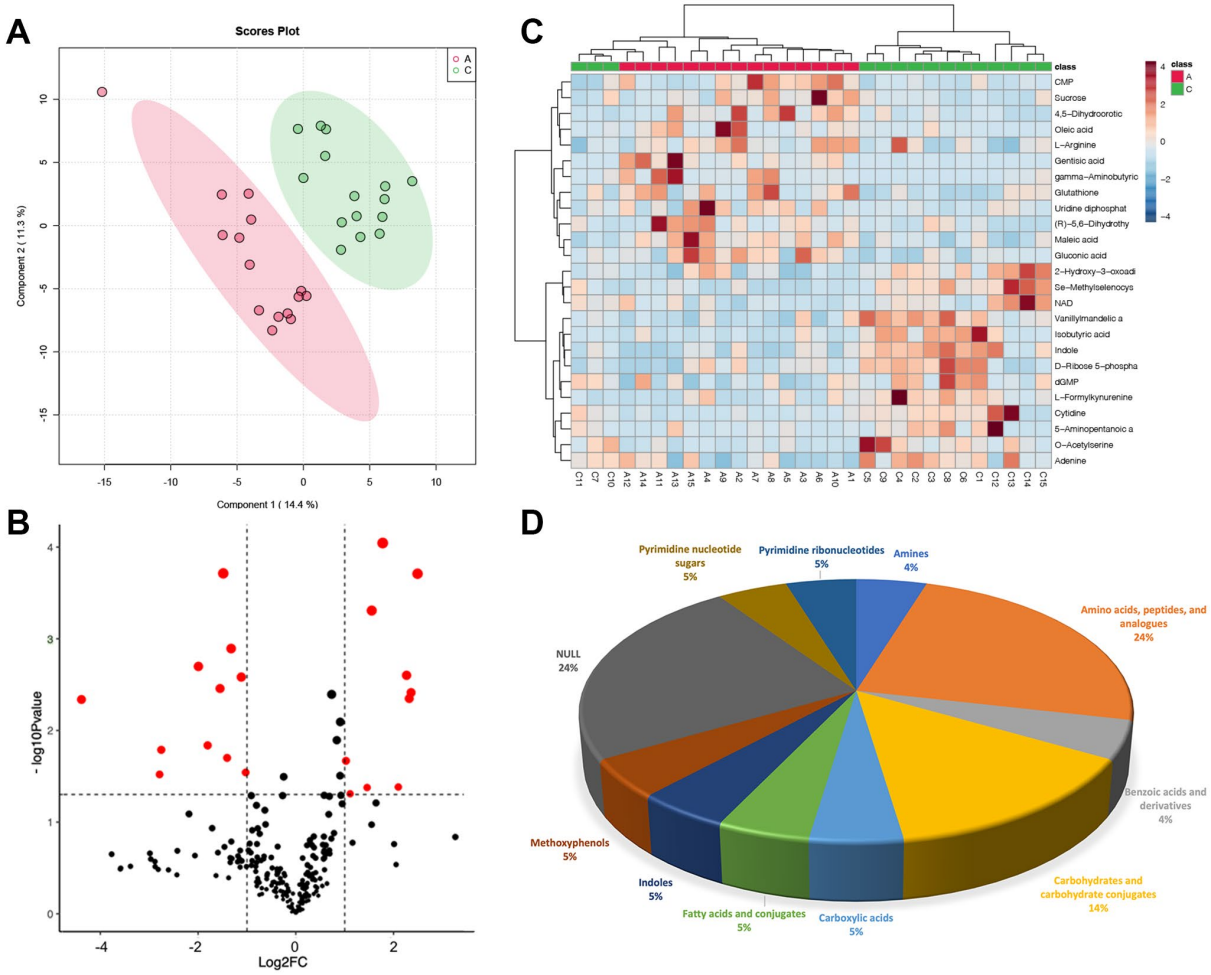
**Metabolic differences in GC between group A and group C.** (**A**) PLSDA score plot; (**B**) volcano plot of differential metabolites, each point represents a differential metabolite, the horizontal coordinate represents the log2 fold change of the metabolite, and the vertical coordinate represents the *P*-value (log10 transformed); (**C**) heatmap of top 25 differential metabolites; (**D**) sub-classification of differential metabolites.

PCA cannot ignore within-group errors, eliminate random errors unrelated to the purpose of the study, and is not conducive to finding differences between groups, therefore, PLSDA was utilized to obtain metabolites that differed significantly between Group A and Group C (Figure 2A). Then, differential metabolites in the two groups were screened, and we got 21 metabolites that differed greatly between Group A and Group C (Figure 2B): 11 were up-regulated and 10 were down-regulated (Supplementary Table 1). Heatmap showed the top 25 differential metabolites (Figure 2C), among them, five metabolites (24%) belong to amino acids, peptides, and analogs, three metabolites (14%) belong to carbohydrates and carbohydrate conjugates, and five metabolites (24%) cannot be classified to any sub-class, the rest of metabolites belonged to amines (4%), benzoic acids and derivatives (4%), carboxylic acids (5%), fatty acids and conjugates (5%), indoles (5%), methoxyphenyl (5%), pyrimidine nucleotide sugars (5%) and pyrimidine ribonucleotides (5%), respectively (Figure 2D).

### Metabolic differences in FF between group A and group C

**Figure 3:**
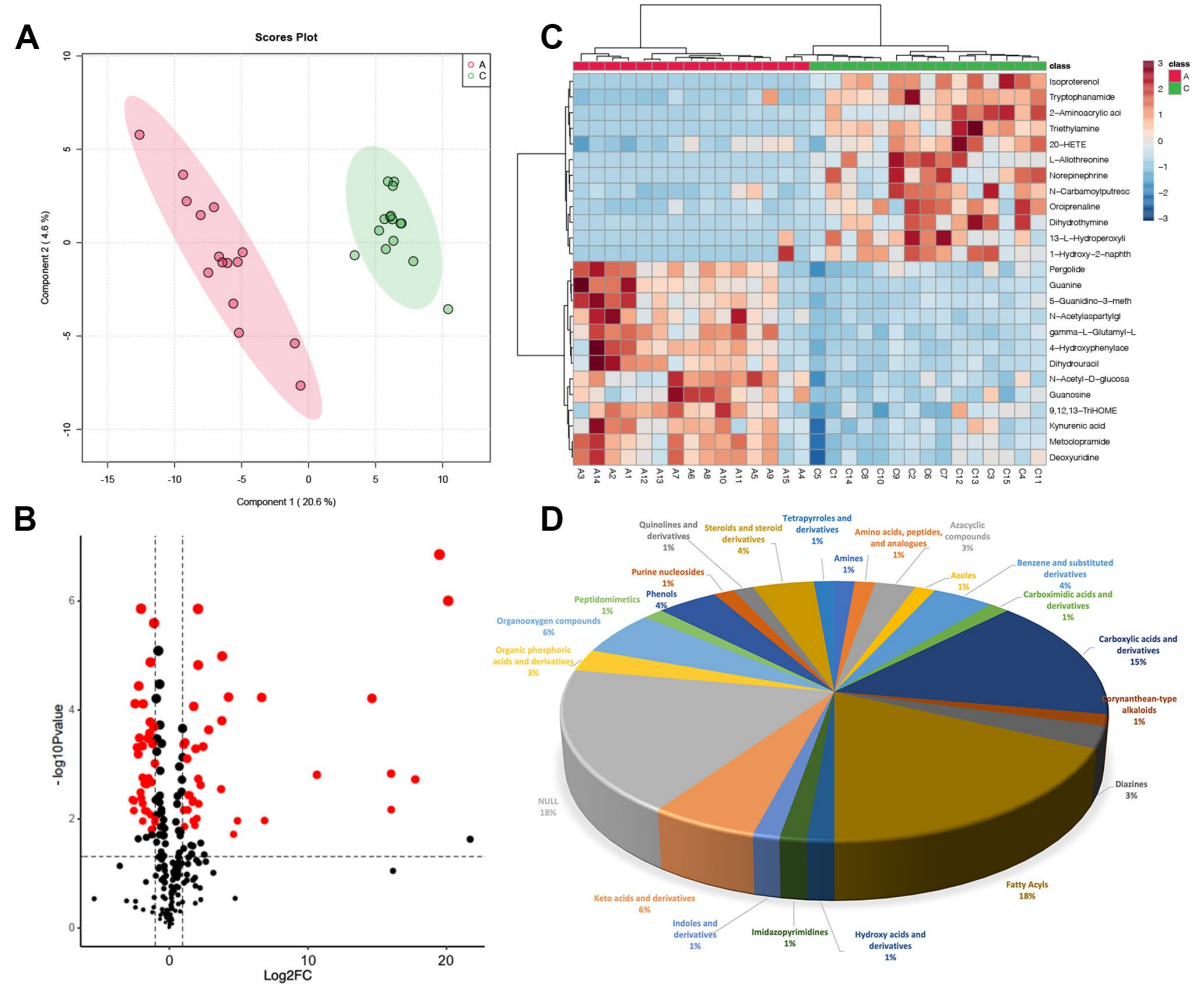
**Metabolic differences in FF between group A and group C.** (**A**) PLSDA score plot; (**B**) volcano plot of differential metabolites, each point represents a differential metabolite, the horizontal coordinate represents the log2 fold change of the metabolite, and the vertical coordinate represents the *P*-value (log10 transformed); (**C**) heatmap of top 25 differential metabolites; (**D**) sub-classification of differential metabolites.

Similarly, we used PLSDA to screen the differences between Group A and Group C (Figure 3A). Finally, a total of 72 metabolites were selected based on the threshold that we set, volcano plot was used for visualization. Heatmap showed the top 25 differential metabolites (Figure 3B, 3C), among them, 37 were down-regulated, and 35 were up-regulated in Group C, and palmitic acid was up-regulated (Supplementary Table 2). Of the 72 differential metabolites, 13 metabolites (18%) belong to fatty acyls and 11 metabolites (15%) belong to carboxylic acids and derivatives. Moreover, keto acids and derivatives and organooxygen compounds each made up 6% of the total, benzene and substituted derivatives, phenols and steroids, and steroid derivatives accounted for 4%, respectively (Figure 3D).

### KEGG metabolic pathway of differential metabolites in FF and GC

To further analyze the function of these differential metabolites, we performed a KEGG enrichment analysis. Through metabolic pathway analysis (MetPA), the associated metabolic pathways of the two groups of differential metabolites were analyzed, the hypergeometric test was used for data analysis and the pathway topology is the relative-betweenness centrality. The pathway impact value represents the importance of the pathway by summing the importance measures of the differential metabolites enriched to the pathway and is divided by the sum of the importance measures of all metabolites on the pathway, which is the pathway impact value. In brief, the larger the value, the more important the pathway.

**Figure 4:**
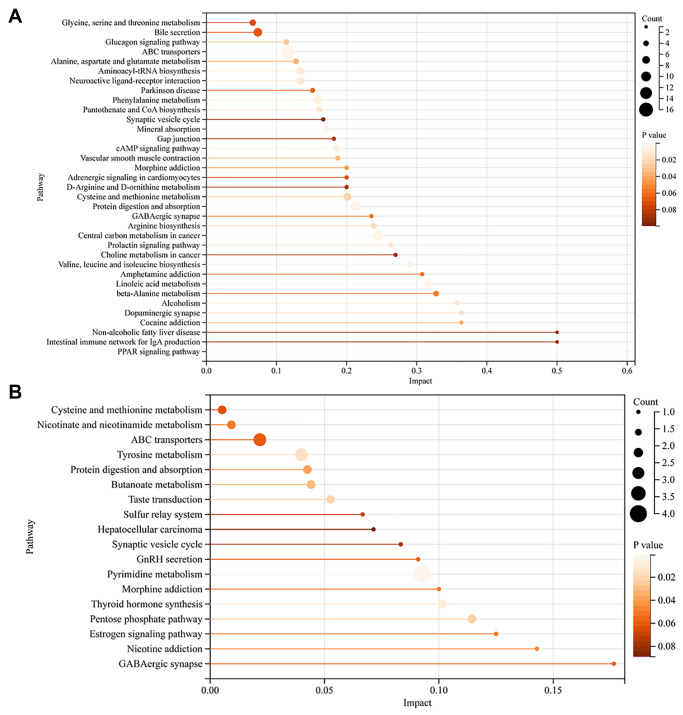
**KEGG metabolic pathway of differential metabolites in FF and GC.** (**A**) KEGG pathway enrichment analysis of FF metabolites; (**B**) KEGG pathway enrichment analysis of GC metabolites. The vertical coordinate represents the corresponding pathway and the horizontal coordinate represents the impact value of the pathway. The size of the dot represents the number of differential metabolites in the target metabolic pathway, the larger the dot, the greater the number of metabolites enriched into that pathway. *P*-values are represented by color, the darker the color, the smaller the *p*-value.

For FF, there were 126 pathways enriched for the differential metabolites, among them, the *P*-value for 35 metabolic pathways was less than 0.1, and 22 pathways had *P*-value < 0.05. Interestingly, we found that the PPAR signaling pathway, linoleic acid metabolism, arginine biosynthesis, cysteine and methionine metabolism, cAMP signaling pathway, pantothenate and CoA biosynthesis, alanine, and aspartate and glutamate metabolism were also significantly enriched (Figure 4A).

**Figure 5:**
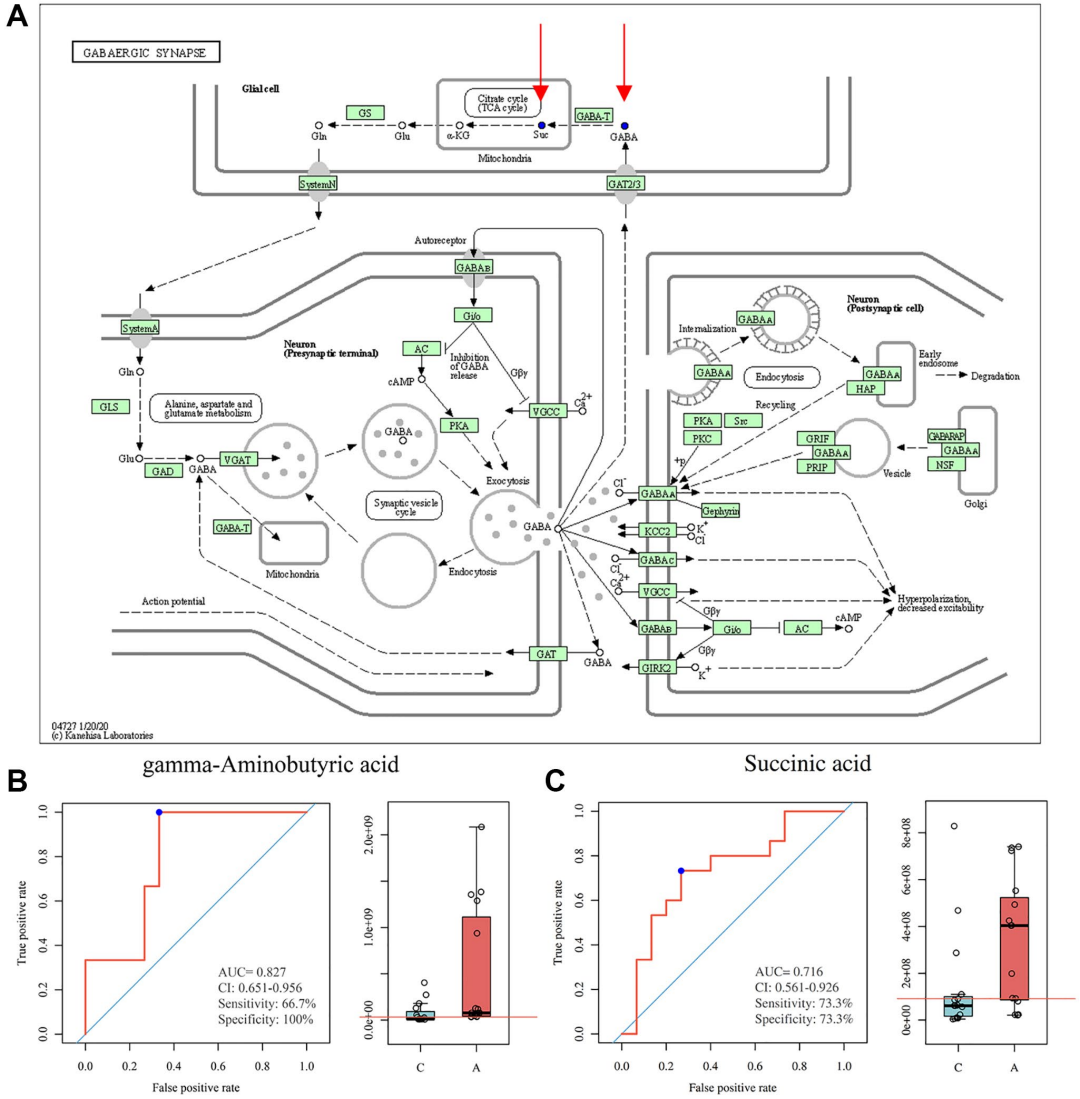
**GABAergic synapse pathway model and ROC curve analysis.** (**A**) KEGG pathway for GABAergic synapse, blue dots represent down-regulated metabolites (https://www.genome.jp/kegg/); (**B**) ROC curve analysis for GABA; (**C**) ROC curve analysis for succinic acid.

For GC, 43 metabolic pathways were enriched, 18 pathways had a *P*-value < 0.1, and 8 pathways had a *P*-value < 0.05 (Figure 4B). Notably, the pentose phosphate pathway, nicotinate and nicotinamide metabolism, GnRH secretion, and estrogen signaling pathway were also involved in the enrichment of differential metabolites. We then took the intersection of the pathways notably enriched by the two sets of samples and found that 6 pathways were co-enriched by the differential metabolites of FF and GC: protein digestion and absorption, ABC transporters, GABAergic synapse, morphine addiction, synaptic vesicle cycle, cysteine, and methionine metabolism. Among them, we noticed that the pathway, GABAergic synapse, had a high impact value in both FF and GC enrichment. After screening the metabolites involved in this pathway, we found that GABA was down-regulated in GC, while its downstream metabolite succinic acid was down-regulated in FF (Figure 5A). Further ROC curve analysis was performed on these two metabolites and results showed that they all had a favorable predictive value (Figure 5B, 5C).

## DISCUSSION

In this study, samples from women in the younger and AMA groups were analyzed by LC-MS/MS analysis, 72 and 21 differential metabolites were screened in FF and GC, respectively. After metabolic pathway enrichment analysis of these differential metabolites, we found that the GABAergic synapse pathway is one of the pathways shared by both and that the levels of two metabolites, GABA and succinic acid, were significantly lower in the advanced age group, and the ROC curve results suggest that they may be potential targets for detecting follicular health and ameliorating the effects of aging on it.

Several studies have shown that age plays a crucial role in maintaining a woman’s fertility. The decrease in female fertility is a reflection of the decrease in oocyte quality and quantity [1, 14]. The development and maturation of the oocyte cannot be separated from the supportive role of the FF and GC, FF contains a variety of nutrients, hormones, and growth factors that are essential for the maintenance of meiotic arrest and ovulation [15], while GC is a layer of wall cells on the follicular surface that plays a role in hormone synthesis to maintain the normal function of the ovary [16]. Therefore, it is of clinical significance to understand the metabolic changes of FF and GC during aging to find therapeutic targets.

In our study, GABA was found to be downregulated in GC of AMA women, we speculate that this is closely correlated with the diminished ovarian function of these patients in the AMA group, although the levels of AMH and FSH, which are indicators of ovarian reserve, were not significantly different in these populations compared to the younger population, they had significantly poorer ovarian responsiveness (total Gn dosage) as well as ART outcomes (number of oocytes obtained, MII oocytes, and 2PN embryos). At the same time, the reason for the lack of significant differences in hormone levels between younger and AMA groups is that to keep the ovulation induction protocol used in the study the same protocol, we chose the follicular phase long-acting protocol. This protocol has certain requirements for ovarian function so patients with extremely poor ovarian function are excluded. GABA is the main inhibitory neurotransmitter in the mammalian central nervous system and has been demonstrated to be closely associated with depression and epilepsy [17, 18]. Because of its capability to modulate neuronal excitability and exert neuroprotective effects, it is clinically used as a sedative and antidepressant [19]. Recently, the role of GABA beyond the central nervous system has raised widespread interest. As reported in one study, the levels of antioxidant enzymes (catalase, superoxide dismutase, and glutathione reductase) in PCOS model mice were restored to varying degrees, and reactive oxygen species (ROS) levels were significantly decreased after feeding GABA to letrozole-induced PCOS model mice [20]. Intracellular ROS is a by-product of the oxidative respiratory chain in mitochondria, which performs a role in regulating cell signaling activities. However, excessive ROS can cause oxidative stress and damage to cells, which in turn can lead to aging and various diseases in the human body, among which, the accumulation of ROS in the ovaries would cause ovarian aging and impair oocyte quality, resulting in female infertility [21, 22]. Many studies have shown the efficacy of GABA as a natural antioxidant in a variety of diseases and metabolic disorder states. Zhu et al. found that GABA inhibited oxidative stress damage to the vascular endothelium caused by hydrogen peroxide-activated NF-κB and caspase-3 signal pathways [23], Lee et al. discovered that GABA ameliorated oxidative stress and endoplasmic reticulum stress in obese mice and reduced the NADP+/NADPH ratio (correlated with ROS production) [24], and Gérald et al. showed that GABA increased the expression of NAD+ and SIRT1 and thus protected pancreatic β-cells [25], while the increase of NAD+ in the ovary significantly improved egg quality and fecundity in aging mice [26]. In addition, the addition of GABA to the feed of hens can also regulate FSH, LH, and thyroid hormone levels increase their egg production, and improve egg quality [27].

As mentioned above, the aging of female ovaries is positively correlated with ROS level, and a decrease in the concentration of GABA as an antioxidant may lead to the accumulation of ROS and thus impair ovarian function, including the quality and quantity of oocytes. Many antioxidants have been used to restore aging ovaries, such as coenzyme Q10, and melatonin [21], our study suggests that GABA may also be a therapeutic tool, but the specific mechanism needs to be further investigated.

As an intermediate during the tricarboxylic acid (TCA) cycle, succinate has an essential part to play in the production of adenosine triphosphate (ATP) in mitochondria, and it is also an endogenous metabolite of the GABA-succinic acid shunt [28]. In humans, succinate has a central sedative effect. Recently, researchers discovered that it can slow down cellular senescence by decreasing Bax expression and upregulating Bcl-2, SIRT2, and Foxo3a [29]. Additionally, in coordination with methionine, it can reduce the damage of vascular endothelium by oxidative stress [30]. We found that succinic acid was down-regulated in FF, which may reflect the TCA cycle was inhibited and resulted in an alteration of mitochondrial energy metabolism. There are few studies on the role of succinic acid in ovarian aging, and our results suggested that succinic acid may be a potential metabolic target for ovarian aging, which still needs to be further investigated in the future.

Fatty acids are potential factors influencing oocyte maturation and subsequent embryonic development; in addition to being a source of energy, they also participate in cell membrane formation and signal transduction to regulate physiological activities [31]. It’s reported that palmitic acid can induce lipotoxicity and thus inhibit the proliferation of bovine granulosa cells and promote apoptosis, thus impairing the maturation of oocytes and development of embryo, besides, it can cause metabolic inflammation and insulin resistance, which are closely related to the development of PCOS [8, 32]. In our study, 18% of differential metabolites in FF belonged to fatty acyls, of which palmitic acid was up-regulated. Correspondingly, these patients got fewer oocytes and their mature oocytes were much fewer than younger patients. How to reduce the palmitic acid concentration in the follicular fluid and thus improve egg quality in advanced women is an urgent issue to be considered.

Several limitations existed in our study. Firstly, the sample size was not large enough and it was a single-center study, which may lead to bias in our conclusions. Secondly, the patients in this study were given the same protocol, but the ovulation drugs used were from different companies, so the effect of the drugs on them cannot be completely excluded.

In conclusion, our study demonstrated that ovarian function declines with aging, although the test indicators did not show much difference with younger patients, AMA patients have poor reproductive outcomes. Metabolomics revealed succinic acid could be a potential target for treatment, and GABA may delay ovarian aging and improve ovarian function through its antioxidant properties, which may be a future direction of clinical treatment.

## Supplementary Materials

Supplementary Tables
