# Supplementary material for: Identification of metabolomic changes and potential therapeutic targets during ovarian aging
Source: Aging (Albany NY). 2024 Oct 9;16(19):12893–908. doi: 10.18632/aging.206119 (PMC11501388; doi:10.18632/aging.206119)
Supplement: Supplementary Tables [file aging-16-206119-s001.pdf]

SUPPLEMENTARY TABLES

Supplementary Table 1. Metabolic differences in GC between group A and group C.

Metabolite	T	*P*-value	Log_{10} *P*-value	FDR	VIP	Foldchange	Log_2 FC
Indole	–4.5677	9.04E-05	4.044	0.016995	2.8654	3.428414701	1.778
Maleic acid	4.2874	0.00019359	3.7131	0.016995	2.7606	0.357359826	–1.485
Se-Methylselenocysteine	–4.2841	0.00019535	3.7092	0.016995	2.7593	5.618608832	2.491
Vanillylmandelic acid	–3.9404	0.00049288	3.3073	0.03216	2.619	2.934616739	1.554
CMP	3.5798	0.0012801	2.8928	0.06682	2.4571	0.399632338	–1.324
4,5-Dihydroorotic acid	3.4071	0.0020054	2.6978	0.085415	2.3739	0.251218409	–1.993
O-Acetylserine	–3.3207	0.0025045	2.6013	0.085415	2.3309	4.810236183	2.267
Gluconic acid	3.3033	0.0026181	2.582	0.085415	2.3221	0.461510061	–1.116
Glutathione	3.1904	0.0034891	2.4573	0.092316	2.2642	0.34135518	–1.551
Cytidine	–3.1497	0.0038663	2.4127	0.092316	2.2429	5.125313925	2.358
Isobutyric acid	–3.0903	0.0044877	2.348	0.092316	2.2114	4.994256605	2.321
Gentisic acid	3.0805	0.0045981	2.3374	0.092316	2.2062	0.047821721	–4.387
(R)-5,6-Dihydrothymine	2.6047	0.014556	1.837	0.23744	1.9366	0.286270468	–1.805
gamma-Aminobutyric acid	2.5564	0.016287	1.7882	0.25005	1.9076	0.148290947	–2.754
Sucrose	2.4686	0.019934	1.7004	0.28904	1.8539	0.377429704	–1.406
NAD	–2.4361	0.021462	1.6683	0.29483	1.8338	2.036908787	1.027
Uridine diphosphate glucuronic acid	2.3062	0.028716	1.5419	0.36332	1.752	0.490942118	–1.027
Oleic acid	2.2837	0.030179	1.5203	0.36332	1.7376	0.144795332	–2.788
5-Aminopentanoic acid	–2.1376	0.041418	1.3828	0.43813	1.6425	4.278440936	2.098
L-Formylkynurenine	–2.1315	0.041966	1.3771	0.43813	1.6384	2.748385324	1.459
Fructose 1,6-bisphosphate	–2.0575	0.049058	1.3093	0.44056	1.5892	2.159547359	1.111

Supplementary Table 2. Metabolic differences in FF between group A and group C.

Metabolite	T	*P*-value	Log_{10} *P*-value	FDR	VIP	Foldchange	Log_2 FC
Isoproterenol	–6.9644	1.43E-07	6.8458	3.34E-05	1.8807	7.37E+05	19.492
Triethylamine	–6.2224	1.01E-06	5.9966	8.29E-05	1.7993	1.14E+06	20.126
gamma-L-Glutamyl-L-2-aminobutyrate	6.0979	1.41E-06	5.8518	8.29E-05	1.7839	0.247286036	–2.016
Tryptophanamide	–6.0953	1.42E-06	5.8487	8.29E-05	1.7836	4.270401845	2.095
N-Acetyl-D-glucosamine	5.8721	2.58E-06	5.5879	0.00012089	1.7546	0.474585924	–1.076
2-Aminoacrylic acid	–5.3574	1.04E-05	4.981	0.00034924	1.6805	14.21039917	3.829
4-Hydroxyphenylacetaldehyde	5.2649	1.34E-05	4.8713	0.00039337	1.6659	0.395350676	–1.339
Orciprenaline	–5.2202	1.52E-05	4.8184	0.00039499	1.6588	4.282288455	2.099
N-Acetylaspartylglutamic acid	4.8964	3.68E-05	4.434	0.00078318	1.6042	0.220191567	–2.184
Dihydrothymine	–4.7247	5.89E-05	4.2302	0.00097657	1.5731	19.52476717	4.288
Norepinephrine	–4.719	5.98E-05	4.2234	0.00097657	1.5721	102.5188891	6.68
L-Allothreonine	–4.7054	6.21E-05	4.2072	0.00097657	1.5695	2.54E+04	14.632
Guanine	4.6224	7.78E-05	4.1088	0.0010808	1.5539	0.183921576	–2.443
Dihydrouracil	4.6191	7.85E-05	4.105	0.0010808	1.5533	0.275095596	–1.862
N-Carbamoylputrescine	–4.5819	8.69E-05	4.0609	0.00113	1.5461	3.433476395	1.78

13-L-Hydroperoxylinoleic acid	–4.3593	0.0001593	3.7978	0.001962	1.5018	13.99678074	3.808
5-Guanidino-3-methyl-2-oxopentanoate	4.3391	0.00016829	3.7739	0.001969	1.4977	0.389878748	–1.359
9,12,13-TriHOME	4.2617	0.00020756	3.6829	0.0022076	1.4815	0.470278405	–1.089
1-Hydroxy-2-naphthoate	–4.2161	0.00023479	3.6293	0.0022892	1.4719	7.259001161	2.86
Guanosine	4.1625	0.00027136	3.5665	0.0025399	1.4603	0.380474071	–1.395
Quadrone	4.089	0.00033087	3.4803	0.0028615	1.4442	0.227603787	–2.136
Dehydroepiandrosterone	4.082	0.00033712	3.4722	0.0028615	1.4427	0.346860909	–1.528
L-Histidine trimethylbetaine	–4.0174	0.0004011	3.3968	0.0031904	1.4282	2.218721573	1.15
9-Riburonosyladenine	3.9965	0.00042419	3.3724	0.0031904	1.4235	0.439251515	–1.187
Linoleic acid	–3.986	0.00043629	3.3602	0.0031904	1.4211	2.089951513	1.064
Quinacrine	3.9617	0.00046558	3.332	0.0032758	1.4156	0.266865927	–1.906
Sorbitol	–3.9535	0.00047597	3.3224	0.0032758	1.4137	5.502366017	2.461
Vitamin D3	3.9396	0.0004939	3.3064	0.0033021	1.4105	0.202691746	–2.303
Ketoleucine	–3.9189	0.00052201	3.2823	0.003393	1.4057	3.788022274	1.922
L-Phenylalanine	3.8321	0.000658	3.1818	0.0040519	1.3854	0.214924347	–2.219
Biliverdin	–3.761	0.00079453	3.0999	0.004648	1.3684	2.479297863	1.31
15-Deoxy-d-12,14-PGJ2	3.6853	0.00097045	3.013	0.0055387	1.3499	0.493315574	–1.02
Carnosine	–3.521	0.0014926	2.8261	0.007938	1.3085	6.58E+04	16.007
Ritodrine	–3.5015	0.0015702	2.804	0.0081651	1.3034	1616.448985	10.659
Butyryl-L-carnitine	3.4557	0.0017688	2.7523	0.0089978	1.2915	0.267301061	–1.904
4-Quinolinecarboxylic acid	3.4448	0.0018192	2.7401	0.0090147	1.2886	0.361807591	–1.467
12-Keto-tetrahydro-leukotriene B4	–3.4362	0.0018604	2.7304	0.0090147	1.2864	4.30200043	2.106
14alpha-Hydroxy-5beta-cholest-7-ene-3,6-dione	–3.4273	0.0019037	2.7204	0.0090147	1.284	2.21E+05	17.752
N-Acetyl-L-2-amino-6-oxopimelate	3.3803	0.002149	2.6678	0.0098602	1.2715	0.415282392	–1.268
Acetylphosphate	3.3568	0.0022829	2.6415	0.010273	1.2652	0.29549081	–1.759
Qing Hau Sau	3.3461	0.0023462	2.6296	0.010359	1.2624	0.325913372	–1.618
Fomepizole	–3.3328	0.0024279	2.6148	0.010521	1.2588	4.853190973	2.279
cis,cis-Muconate	–3.2662	0.002878	2.5409	0.012244	1.2406	13.51460929	3.757
Docosapentaenoic acid (22n-3)	3.2134	0.0032919	2.4826	0.013514	1.226	0.241995983	–2.047
O-Phosphoethanolamine	–3.1679	0.0036926	2.4327	0.014671	1.2132	2.627913699	1.394
Phthalic acid	–3.1641	0.0037283	2.4285	0.014671	1.2122	2.820954047	1.497
2-Oxoarginine	3.1157	0.0042106	2.3757	0.015703	1.1984	0.250909547	–1.995
Adrenic acid	3.0881	0.0045124	2.3456	0.016285	1.1905	0.161561329	–2.63
D-Arabitol	–3.0756	0.0046548	2.3321	0.01642	1.1869	3.329448976	1.736
UMP	3.0716	0.0047016	2.3278	0.01642	1.1858	0.173767985	–2.525
Methyl jasmonate	–3.0515	0.004943	2.306	0.01701	1.18	3.239495934	1.696
9(S)-HPODE	3.0264	0.0052621	2.2788	0.017479	1.1726	0.268643886	–1.897
D-Glucose	–3.0196	0.0053507	2.2716	0.017479	1.1707	4.406062742	2.14
3-Aminopentanedioate	–2.9192	0.006853	2.1641	0.021373	1.1409	6.62E+04	16.015
Palmitic acid	–2.9144	0.0069352	2.1589	0.021373	1.1395	2.553952241	1.353
Alpha-dimorphecolic acid	–2.9136	0.0069479	2.1581	0.021373	1.1393	2.082075413	1.059
Succinic acid	2.9052	0.0070926	2.1492	0.021373	1.1367	0.29856985	–1.744
Adenosine	2.9034	0.0071244	2.1473	0.021373	1.1362	0.172532781	–2.536
L-Isoleucine	2.8921	0.0073237	2.1353	0.021422	1.1328	0.333055787	–1.587

2-Ketobutyric acid	2.8326	0.0084619	2.0725	0.024147	1.1147	0.398596939	–1.327
(R)-3-Hydroxybutyric acid	–2.7652	0.009954	2.002	0.027729	1.0939	4.006570776	2.003
5a-Pregnane-3,20-dione	2.743	0.010498	1.9789	0.028564	1.087	0.498753117	–1.004
Glycochenodeoxycholic acid	–2.7297	0.010837	1.9651	0.028864	1.0828	118.2718123	6.886
L-Asparagine	–2.7255	0.010944	1.9608	0.028864	1.0815	30.61474406	4.937
Oxoadipic acid	–2.7229	0.011012	1.9581	0.028864	1.0807	3.349186148	1.744
(S)-4-Amino-5-oxopentanoate	2.7195	0.011102	1.9546	0.028864	1.0797	0.269063122	–1.894
Mitragynine	2.703	0.011546	1.9376	0.029366	1.0745	0.494951495	–1.015
Pyroglutamic acid	–2.6426	0.013317	1.8756	0.033507	1.0553	3.647372068	1.867
Alpha-Linolenic acid	–2.6225	0.01396	1.8551	0.034387	1.0489	2.172165867	1.12
(R)-2,3-Dihydroxy-isovalerate	2.5819	0.01535	1.8139	0.036653	1.0358	0.440295879	–1.184
Myristic acid	2.5664	0.015915	1.7982	0.037616	1.0307	0.41322314	–1.276
L-4-Hydroxyphenylglycine	–2.4813	0.019362	1.7131	0.043825	1.0028	25.2016129	4.656
